# Sweet food preference in amyotrophic lateral sclerosis

**DOI:** 10.1136/practneurol-2016-001554

**Published:** 2017-01-10

**Authors:** Martin R Turner, Kevin Talbot

**Affiliations:** Nuffield Department of Clinical Neurosciences, University of Oxford, Oxford, UK

**Keywords:** MOTOR NEURON DISEASE, ALS, DEMENTIA

**Figure 1:**
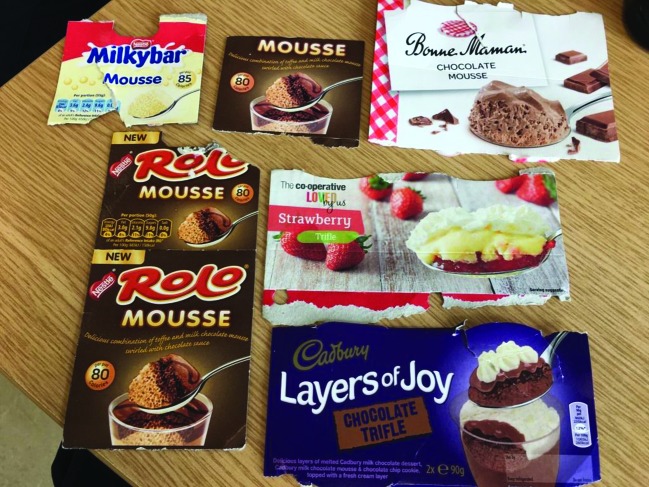
Packaging cut-outs carried by the patient to indicate her preferred food in the absence of speech. It was noted that all of them were confectionery items, consistent with observations that patients with amyotrophic lateral sclerosis may develop an exaggerated preference for sweet foods as a manifestation of frontal lobe pathology.

An elderly female developed anarthria with prominent emotionality over an 18-month period before specialist neurological assessment. Although tongue electromyography was normal, her corticobulbar signs were consistent with amyotrophic lateral sclerosis (ALS), a pattern that, in the absence of ­functional impairment outside of speech and swallowing, is appropriately termed progressive bulbar palsy. Such patients, often elderly females, may remain ambulant and independent for many months, sometimes years, despite typically rapid anarthria.^1^ Electromyography may be insensitive to denervation, even when genioglossus is sampled, and this can contribute to diagnostic delay in patients with corticobulbar presentations of ALS who are frequently referred to ‘TIA’ or ENT clinics.^2^ When asked about her nutritional state, she revealed a collection of pictures of her favourite foods carried in her handbag to facilitate communication (figure 1).

ALS has pathological overlap with frontotemporal dementia through the common feature of cytoplasmic inclusions containing TDP-43. A hexanucleotide expansion in *C9orf72* is associated with both ‘pure’ and mixed cases of ALS and frontotemporal dementia which may occur within the same pedigree.^3^ Overt dementia is not common in ALS (up to 15% in population-based studies), and is typically an early feature coincident with motor signs when it occurs. However, up to 50% of patients with ALS show a spectrum of more subtle cognitive and behavioural change, though most of these will not go on to develop dementia during the course of their disease. There have been criteria developed to reflect this broader phenotypic range of extramotor involvement in ALS.^4^

An acquired preference for sweet foods, often with a narrowed repertoire, is included in the criteria for frontotemporal dementia. In ALS cases, it is a clue to frontotemporal involvement, and part of an emerging array of metabolic disturbances common to both disorders.^5^ It should prompt more detailed neuropsychological assessment if there are wider concerns about behaviour or capacity.
